# Gastrectomy is Associated with an Increased Risk of Pyogenic Liver Abscess: A 13-Year Nationwide Cohort Study

**DOI:** 10.1038/srep33788

**Published:** 2016-09-27

**Authors:** Ming-Shian Tsai, Cheng-Li Lin, Long-Bin Jeng

**Affiliations:** 1 Division of General Surgery, Department of Surgery, E-Da Hospital and I-Shou University, Kaohsiung, Taiwan; 2 Bariatric and Metabolic Surgery Center, E-Da Hospital and I-Shou University, Kaohsiung, Taiwan; 3 Management Office for Health Data, China Medical University Hospital, Taichung, Taiwan; 4 College of Medicine, China Medical University, Taichung, Taiwan; 5 Graduate Institute of Clinical Medical Science and School of Medicine, College of Medicine, China Medical University, Taichung, Taiwan; 6 Department of Surgery, Organ Transplantation Center, China Medical University Hospital, Taichung, Taiwan

## Abstract

Whether patients who have undergone gastrectomy are at a high risk of developing pyogenic liver abscess (PLA) remains debatable. From the inpatient claims records of Taiwan’s National Health Insurance Research Database, we identified 33 834 patients with a history of 2000–2010 and135 336 controls without a history of gastrectomy. The 2cohorts were matched by age, sex, and admission year and followed-up until the end of 2011 for estimating the risk of PLA. Overall, the incidence of PLA was 3.5-fold higher in the gastrectomy cohort than in the control cohort (21.6 vs 5.76 per 10 000 person-y). The adjusted hazard ratio (aHR) for the gastrectomy cohort obtained using the multivariate Cox proportional hazards regression model was 3.08 (95% confidence interval [CI] = 2.60–3.64). An elevated post gastrectomy PLA risk was observed in both men and women. Age-specific data revealed that the aHR for the gastrectomy cohort, compared with the control cohort, was the highest in patients younger than 50 years (aHR = 5.16, 95% CI = 2.96–9.01). An addition analysis showed that the gastrectomy cohort exhibited an elevated PLA risk regardless of whether the patients underwent total or partial gastrectomy. Patients with a history of gastrectomy exhibit a high risk of PLA.

Gastrectomy is a widely used treatment choice for many diseases, including morbid obesity, peptic ulcer diseases, and gastric neoplasm. Short-term complications of gastrectomy include leakage, intra-abdominal and/or gastrointestinal hemorrhage, and bowel obstruction. In addition, gastrectomy carries the risks of certain long-term complications, such as anastomotic ulcer^1^, cholelithiasis^2^,^3^,^4^, and anemia^5^,^6^. However, whether patients who have under gone gastrectomy exhibit increased risks of bacterial infection in their digestive system remains unclear.

Gastric acid is a crucial mechanism against digestive system infection^7^,^8^,^9^,^10^. Therefore, medical and surgical treatments that decrease gastric acid secretion may increase the risk of digestive tract infection. For example, the use of gastric acid-suppressing agents is associated with an increased risk of *Clostridium difficile* infection^11^. Moreover, gastrectomy has been associated with enterocolitis^10^,^11^,^12^,^13^. In severe outbreaks of *E. coli*-related colitis, gastrectomy is considered an independent risk factor for infection^10^.

Without prompt recognition and treatment, pyogenic liver abscess (PLA) can be fatal^14^. PLA is commonly associated with an underlying gastrointestinal pathology^15^. Hematogenous propagation of pathogens from the digestive tract to the liver was proposed as a pathogenic factor for PLA^16^. Theoretically, various conditions causing intestinal infection may increase the PLA risk^17^,^18^,^19^, and clinically identifying the underlying etiology is an integral part of PLA management.

Based on our research, however, the association between gastrectomy and PLA has not yet been investigated. We hypothesize that patients who have undergone gastrectomy may have a higher risk of PLA than that of patients without a history of gastrectomy. In the present study, we explored the association between gastrectomy and PLA by analyzing data from Taiwan’s National Health Insurance Research Database (NHIRD), which contains deidentified medical claims data from 99% of the 23 million residents of Taiwan.

## Results

Our study included 33 834 patients with a history of gastrectomy and 135 336 controls. In the gastrectomy cohort, 55.1% of the patients were ≥65 years old, and 67.1% were men (Table 1). The mean ages in the gastrectomy and control cohorts were 64.7 (standard deviation [SD] = 14.2) and 64.0 (SD = 14.4) years, respectively. Comorbidities were more prevalent in the gastrectomy cohort than in the control cohort (all *P *< 0.001). The median follow-up duration for the control cohort was 5.30 (range = 0.003–12.0) years, approximately 3 years more than that for the gastrectomy cohort (2.28 years; range = 0.003–12.0).

**Figure 1:**
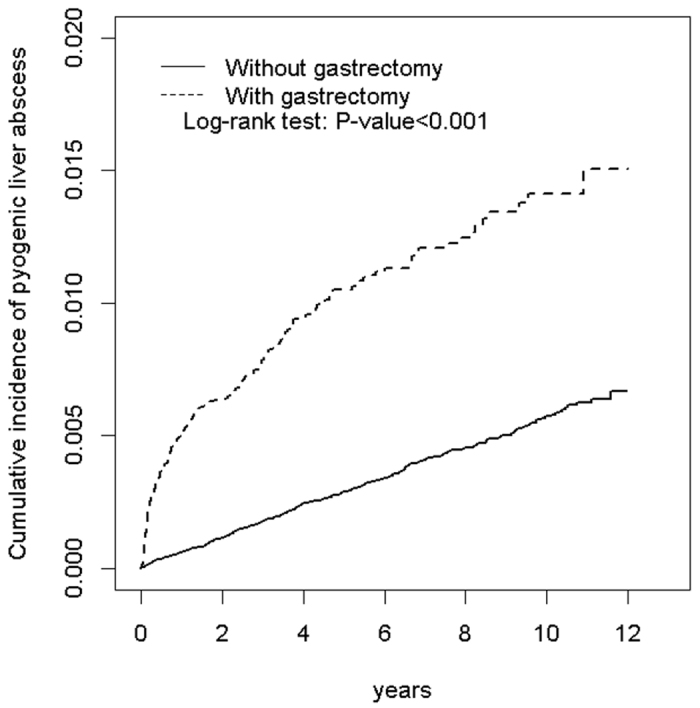
Cumulative incidence of pyogenic liver abscess for patients with and without a history of gastrectomy.

The overall incidence of PLA was 3.5-fold higher in the gastrectomy cohort than in the control cohort (21.6 vs 5.76 per 10 000 person-y, respectively; Table 2). After adjustment for age, sex, and comorbidity, patients with a history of gastrectomy were associated with an increased risk of PLA compared with those without a history of gastrectomy (adjusted HR [aHR] = 3.08, 95% CI = 2.60–3.64). The Kaplan–Meier analysis revealed that the cumulative incidence curves of PLA were significantly higher in the gastrectomy cohort than in the control cohort by 0.84% (log-rank test *P *< 0.001; Fig. 1). Moreover, the two curves of PLA incidence separated gradually during the 12-year follow-up period (Fig. 1), suggesting the association between gastrectomy and PLA was not only related to short-term effects.

We then further analyzed the risk of PLA stratified by sex, age and the presence of comorbidities. The risk of PLA was significantly higher in patients with a history of gastrectomy than in patients without a history of gastrectomy when stratified by sex (aHR = 2.98, 95% CI = 2.18–4.08 for women; aHR = 3.11, 95% CI = 2.55–3.79 for men), age (aHR = 5.16, 95% CI = 2.96–9.01 for patients aged ≤49; aHR = 2.59, 95% CI = 1.89–3.55 for patients aged 50–64; aHR = 3.06, 95% CI = 2.48–3.79 for patients aged ≥65), and comorbidity (aHR = 5.11, 95% CI = 3.94–6.62 for patients without comorbidity; aHR = 2.13, 95% CI = 1.75–2.60 for patients with comorbidities).

Table 3 illustrates the rates and hazard ratios of PLA associated with different extent of gastrectomy, i.e. partial and total gastrectomy. Patients who had undergone total gastrectomy were 3.67-fold more likely to develop PLA than the controls (95% CI = 2.84–4.74). The aHR was higher for patients with a history of partial gastrectomy (aHR = 2.88; 95% CI = 2.39–3.46) than for the controls.

Finally, we investigated the joint effects of gastrectomy and other PLA risk factors on the risk of PLA (Table 4). Compared with the controls without any comorbidity, patients with only cholecystitis had the highest risk of PLA (aHR = 4.26, 95% CI = 1.06–17.2), followed by those with only DM (aHR = 2.94, 95% CI = 2.10–4.12) and those with only cancer (aHR = 2.14, 95% CI = 1.13–4.05; Table 4). Moreover, compared with the controls without any comorbidity, patients with a history of gastrectomy and with 3 or more comorbidities were at a significantly increased risk of PLA (aHR = 6.25, 95% CI = 4.42–8.84), followed by those with 2 comorbidities (aHR = 5.54, 95% CI = 4.03–7.62) and those with one comorbidity (aHR = 5.40, 95% CI = 4.17–7.00).

## Discussion

This nationwide study is the first to report that patients with a history of gastrectomy are at a 3-fold higher risk of PLA compared with patients without a history of gastrectomy. The PLA risk is significantly increased in patients with previous gastrectomy, with an aHR of 3.08 (95% CI = 2.60–3.64) for PLA, after adjustment for confounding factors. Our results, derived from a large-scale epidemiological database, affirm sporadic observations that gastrectomy is associated with PLA^20^,^21^. According to our results, gastrectomy was a risk factor of PLA in patients without other known comorbidities (Table 4). Moreover, in Table 2, we also showed that gastrectomy was associated with PLA in patients both with and without known comorbidities. In a prospective study investigating infection events after transarterial embolization for hepatocellular carcinoma, liver abscess occurred only in patients who had previously undergone gastrectomy^20^. The present study identified that patients with a history of gastrectomy are more vulnerable to PLA.

Because the prognosis of PLA is closely related to prompt diagnosis and management, appropriate screening and management of PLA may be necessary in patients undergoing gastrectomy when there is any clinical suspicion. Moreover, this study raised a potential concern regarding the potential complications of treating morbid obesity through sleeve gastrectomy. More studies are necessary to validate our observations in patients undergoing gastrectomy for different diseases.

Our longitudinal study effectively links gastrectomy and PLA. Unlike cross-sectional and case-control studies, the present study avoids the selection and recall biases and allows to the observation of cumulative incidence of PLA over time (Fig. 1). PLA incidence rises rapidly within the first year after gastrectomy, and the slope of the cumulative incidence curve stabilizes after the first year (Fig. 1), indicating that approximately one-third of the PLAs occurred within the first follow-up year, possibly because several factors affect patient immunity. First, surgery itself alters the immune function^22^. Second, patients undergoing gastrectomy may be relatively malnourished for the first few postoperative months. Third, patients undergoing gastrectomy for malignancy might also receive chemotherapy during the perioperative period. Fourth, gastric perforation may be one major indication for gastrectomy. In this case, gastric perforation may be associated with several immunocompromised conditions, such as steroid use, peritonitis and malnutrition. These factors combine to compromise patient immunity temporarily, thus increasing the risk of infection including PLA.

The curves of cumulative PLA incidence in the gastrectomy and control cohorts gradually diverged during the follow-up period (Fig. 1). The aforementioned surgery-related factors that may predispose the patients to PLA do not completely explain why gastrectomy is associated with the PLA risks observed in the present study. A possible factor distorting our observations is the diagnostic bias: The gastrectomy cohort may undergo diagnostic procedures for PLA more frequently than does the control cohort. In the gastrectomy cohort, abdominal imaging was commonly conducted to detect surgery complications and malignancy recurrence or metastasis, which usually occurs during the first few postoperative years. Therefore, the significant difference in the PLA risk exhibited by the 2cohorts cannot be directly attributed to the diagnostic bias.

PLA incidence in Taiwan ranges from 1.12 to 1.86 events per 10 000 person-years^23^, which is much lower than the incidence reported in our control cohort (5.76 events per 10 000 person-y). As shown in Table 2, the incidence of PLA is higher in men than in women and increases with age. In the present study, men accounted for 67.1% of the 2cohorts, and more than half of the participants were older than 65 years. The difference in the incidence rates may be attributable to differences in the age and sex compositions of the control cohort and the general population in Taiwan.

Multiple factors may predispose patients with a history of gastrectomy to PLA. Gastric acid secretion is an important defense mechanism that regulates the number of ingested bacteria and kills many ingested organisms. Gastrectomy affects this mechanism and consequently increases the number of bacterial flora accessing the gastrointestinal mucosa. A microbiological examination of mucosal specimens in patients who have undergone total gastrectomy revealed significant bacterial flora overgrowth, with streptococci being the most abundant species^24^. In addition, mucosal ulceration commonly occurs at the gastrectomy site and gastrointestinal anastomosis^1^, allowing bacterial breakthrough from the digestive tract to the blood stream; both of these factors might contribute to bacterial infection and liver abscess^9^,^10^,^20^.

Gastrectomy has also been associated with an elevated risk of cholelithiasis^25^,^26^; however, the mechanisms underlying this association are not fully understood. Cholelithiasis is a risk factor for PLA^27^. As high as 36% of men and 19% of women who have undergone total gastrectomy were reported to develop cholelithiasis^26^. Lymph node dissection in the hepatoduodenal ligament, total gastrectomy, and exclusion of the duodenum are risk factors for gallstones after gastrectomy^25^,^26^. We also found that patients who had undergone total gastrectomy exhibited a slightly higher PLA risk than that of those undergoing partial gastrectomy (Table 3). The proportion of PLA after gastrectomy that is attributable to cholelithiasis is worth investigating.

The compromised post gastrectomy nutritional status^28^ is another risk factor for PLA^29^. Malnutrition may alter the immune system and predispose the patient to infection. A comparison of the nutritional status of patients after different extents of gastrectomy revealed that the quantity of some micronutrients reduced after total gastrectomy. No major differences were found in the nutritional status of the total and partial gastrectomy cohorts.

This study has several limitations. First, the NHIRD provides no detailed information on smoking habits, alcohol consumption, physical activity, economic status, or duration and dosage of steroid use, all of which are potentially confounding factors relevant to this study. In particular, the lack of information regarding DM duration and the status of DM control might bias the results. Second, we were unable to validate the diagnoses of PLA and gastrectomy through a chart review. However, the diagnoses are reliable because we included only hospitalized patients whose diagnoses were strictly audited for reimbursement. Last but not least, the differences in follow-up period may be another bias, considering gastrectomy group had a shorter follow-up period. Theoretically, this bias may potentially underestimate the incidence of PLA in the gastrectomy group, since the individuals of gastrectomy group might die before PLA developed.

## Conclusion

In summary, we determined that patients with a history of gastrectomy have an increased risk of PLA. Additional studies on the incidence rates of PLA for patients with gastrectomy history are warranted.

## Methods

### Data Source

Taiwan’s National Health Insurance (NHI) system is a mandatory universal health insurance program that offers comprehensive medical coverage to all Taiwanese residents^20^. The National Health Research Institute (NHRI) of the Ministry of Health and Welfare maintains and releases the NHIRD for use in health service research. To ensure privacy, the NHRI assigns a scrambled, anonymous identification number to the record of each insurant, including sex, birthdate, and registry of medical services; the registry uses the *International Classification of Diseases, Ninth Revision, Clinical Modification* (ICD-9-CM) diagnostic codes. In this study, we used a subset of the NHIRD containing healthcare data, including inpatient claims and the registry of beneficiaries. All NHI datasets are inter linked with the personal identification number of each insurant. This study was approved to fulfill the condition for exemption by the Institutional Review Board (IRB) of China Medical University (CMUH104-REC2-115). The IRB also specifically waived the consent requirement.

### Sampled Participants

**Figure 2:**
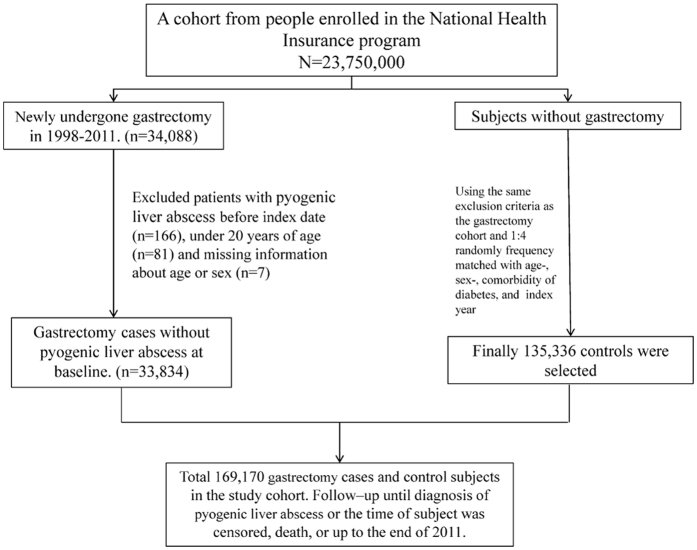
The flow chart shows the study subjects’ selection.

From inpatient claims data, we identified patients older than 20 years who had undergone partial gastrectomy (ICD-9-OP43.5, 43.6, 43.7, 43.8, 43.81, 43.82, 43.89) or total gastrectomy (ICD-9-OP 43.91 and43.99) from January 1, 2000 to December 31, 2010. Patients whose date of birth and sex were missing in the data and those with preexisting PLA (ICD-9-CM 572.0) were excluded. The index date was defined when the ICD 9 codes of gastrectomy was found within the NHIRD database. Usually, the index date was weeks after the patient was discharged or dead, mainly due to the process time for filing the reimbursement. For each gastrectomy patient, four comparisons were randomly selected from the pool of participants without gastrectomy and PLA at the baseline, frequency matched by the year of index date, age (every 5-year span), sex and comorbidity of diabetes (Fig. 2).

### Outcome and Comorbidities

The gastrectomy and control cohorts were followed either until the diagnosis of PLA or until loss to follow-up, death, or December 31, 2011. A diagnosis of one of the following diseases prior to the index date, identified from the hospitalization records, were the considered comorbidities: diabetes mellitus (DM) (ICD-9-CM 250), hypertension (ICD-9-CM 401–405), hyperlipidemia (ICD-9-CM 272), cancer (ICD-9-CM 140–208), chronic obstructive pulmonary disease (COPD) (ICD-9-CM 491, 492, 496), heart failure (ICD-9-CM 428), choledocholithiasis (ICD-9-CM 574), alcoholic liver disease (ICD-9-CM 571.0, 571.1, 571.3), liver cirrhosis (ICD-9-CM 571.2, 571.5, 571.6), cholangitis (ICD-9-CM code 576.1), cholecystitis (ICD-9-CM 575), or pancreatic diseases (ICD-9-CM 577).

### Statistical Analyses

The distributions of categorical demographic characteristics and comorbidities in the gastrectomy and control cohorts were compared using the Chi-square test. The mean age and mean follow-up duration between both cohorts were examined using the Student’s t-test. The cumulative incidence curves of PLA for the 2 cohorts were calculated using the Kaplan–Meier method and compared using the log-rank test. The incidence density rate of PLA in the 2cohorts was calculated for the follow-up period until the end of the study (2011). Univariate and multivariate Cox proportional hazard regressions were used to examine the effect of gastrectomy on the risk of PLA and reported as a hazard ratio (HR) with a 95% confidence interval (CI). The multivariate model was simultaneously adjusted for age, sex, and comorbidities of DM, hypertension, hyperlipidemia, cancer, COPD, heart failure, choledocholithiasis, alcoholic liver disease, liver cirrhosis, cholangitis, cholecystitis, and pancreatic diseases. An additional data analysis was performed to evaluate the combined effects of gastrectomy and PLA-associated risk factors on PLA. All analyses were executed using SAS statistical software (Version 9.4 for Windows; SAS Institute, Inc., Cary, NC, USA); *P* < 0.05 was considered statistically significant.

## Additional Information

**How to cite this article**: Tsai, M.-S. *et al*. Gastrectomy is Associated with an Increased Risk of Pyogenic Liver Abscess: A 13-Year Nationwide Cohort Study. *Sci. Rep*. **6**, 33788; doi: 10.1038/srep33788 (2016).

## Figures and Tables

**Table 1:** Characteristics of patients with and without a history of gastrectomy.

	Gastrectomy				*P*-value
	Yes		No		
	n	%	n	%	
Age, year					0.99
≦49	5825	17.2	23300	17.2	
50–64	9364	27.7	37456	27.7	
≥65	18645	55.1	74580	55.1	
Mean (SD)^#^	64.7	14.2	64.0	14.4	0.001
Sex					0.99
Female	11134	32.9	44536	32.9	
Male	22700	67.1	90800	67.1	
Comorbidity					
Diabetes mellitus	6138	18.1	24552	18.1	0.99
Hypertension	10589	31.3	27215	20.1	<0.001
Hyperlipidemia	1937	5.73	6753	4.99	<0.001
Cancer	8467	25.0	5761	4.26	<0.001
COPD^a^	2747	8.12	7193	5.31	<0.001
Heart failure	1525	4.51	4318	3.19	<0.001
Choledocholithiasis	3190	9.43	4763	3.52	<0.001
Alcoholic liver disease	564	1.67	702	0.52	<0.001
Liver cirrhosis	1549	4.58	1869	1.38	<0.001
Cholangitis	404	1.19	850	0.63	<0.001
Cholecystitis	1470	4.34	1274	0.94	<0.001
Pancreatic diseases	947	2.80	1367	1.01	<0.001
Renal disease	2753	8.14	5501	4.06	<0.001
Diverticulosis	397	1.17	681	0.50	<0.001
Appendicitis	436	1.29	1368	1.01	<0.001
IBD^b^	49	0.14	139	0.10	0.04

Chi-square test; ^#^t-test; SD denotes standard deviation.

^a^COPD = chronic obstructive pulmonary disease; ^b^IBD = Inflammatory Bowel Disease.

**Table 2:** Incidence rates and hazard ratios of pyogenic liver abscess for patients with and without a history of gastrectomy.

Outcome	Gastrectomy						Crude HR †(95% CI)	Adjusted HR^‡^ (95% CI)
	Yes			No				
	Event	PY	Rate^#^	Event	PY	Rate^#^		
All	255	117943	21.6	439	762110	5.76	3.50(3.00, 4.09)***	3.08(2.60, 3.64)***
Sex								
Female	72	40582	17.7	126	254774	4.95	3.33(2.49, 4.46)***	2.98(2.18, 4.08)***
Male	183	77360	23.7	313	507335	6.17	3.59(2.99, 4.31)***	3.11(2.55, 3.79)***
Age, year								
≦49	30	26485	11.3	27	150939	1.79	6.15(3.65, 10.4)***	5.16(2.96, 9.01)***
50–64	72	35728	20.2	132	223033	5.92	3.17(2.37, 4.22)***	2.59(1.89, 3.55)***
≥65	153	55729	27.5	280	388137	7.21	3.54(2.90, 4.31)***	3.06(2.48, 3.79)***
Comorbidity^§^								
No	82	47424	17.3	195	541683	3.60	4.69(3.62, 6.07)***	5.11(3.94, 6.62)***
Yes	173	70519	24.5	244	220527	11.1	2.08(1.71, 2.53)***	2.13(1.75, 2.60)***

PY, person-years; Rate^#^, incidence rate per 10 000 person-years.

Crude HR^†^, relative hazard ratio.

Adjusted HR^‡^, hazard ratio adjusted for age, sex, and comorbidities of DM, hypertension, hyperlipidemia, cancer, COPD, heart failure, choledocholithiasis, alcoholic liver disease, liver cirrhosis, cholangitis, cholecystitis, pancreatic diseases, renal disease, diverticulosis, appendicitis, and IBD.

Comorbidity^§^: Patients with any one of the comorbidities (DM, hypertension, hyperlipidemia, cancer, COPD, heart failure, choledocholithiasis, alcoholic liver disease, liver cirrhosis, cholangitis, cholecystitis, pancreatic diseases, renal disease, diverticulosis, appendicitis, and IBD) were classified as the comorbidity cohort.

***P < 0.001.

**Table 3:** Incidence rates and hazard ratios of pyogenic liver abscess for patients who have undergone partial and total gastrectomy.

Variable	Event	PYs	Rate	Crude HR (95% CI)	Adjusted HR (95% CI)
Control (N = 135336)	439	762110	5.76	1.00	1.00
Gastrectomy					
Partial gastrectomy (N = 24541)	180	90124	20.0	3.27(2.75, 3.89)***	2.88(2.39, 3.46)***
Total gastrectomy (N = 9293)	75	27819	27.0	4.24(3.32,5.42)***	3.67(2.84, 4.74)***

PY, person-years; Rate^#^, incidence rate per 10 000 person-years.

Crude HR^†^, relative hazard ratio.

Adjusted HR^‡^, hazard ratio adjusted for age, sex, and comorbidities of DM, hypertension, hyperlipidemia, cancer, COPD, heart failure, choledocholithiasis, alcoholic liver disease, liver cirrhosis, cholangitis, cholecystitis, pancreatic diseases, renal disease, diverticulosis, appendicitis, and IBD.

***P < 0.001.

**Table 4:** Combined effects of gastrectomy and pyogenic liver abscess-associated risk factors on pyogenic liver abscess.

Variable	N	No. of Events	Rate^#^	Adjusted HR^†^(95% CI)		Adjusted HR^§^(95% CI)	
None	89080	197	3.59	1	(Reference)	1	(Reference)
One risk factor							
Gastrectomy	11903	87	17.6	5.20	(4.04, 6.71)***	3.41	(2.90, 4.02)***
Cholecystitis	208	2	17.3	4.26	(1.06, 17.2)*	1.59	(1.07, 2.36)*
Diabetes mellitus	6735	41	11.4	2.94	(2.10, 4.12)***	2.27	(1.93, 2.68)***
Cancer	2289	10	9.19	2.14	(1.13, 4.05)*	1.13	(0.89, 1.44)
Hypertension	7428	19	5.10	1.05	(0.65, 1.69)		
Hyperlipidemia	706	4	10.4	2.59	(0.96, 6.97)		
COPD^a^	1563	4	5.30	0.98	(0.36, 2.66)		
Heart failure	427	1	5.31	1.03	(0.15, 7.39)		
Choledocholithiasis	968	3	5.53	1.31	(0.42, 4.08)		
Alcoholic liver disease	103	1	17.3	5.15	(0.72, 36.8)		
Liver cirrhosis	233	1	10.7	2.58	(0.36, 18.4)		
Cholangitis	26	0	0.00	—	—		
Pancreatic diseases	118	0	0.00	—	—		
Renal disease	108	0	0.00	—	—		
Diverticulosis	4	0	0.00	—	—		
Appendicitis	1	0	0.00	—	—		
IBD^b^	4	0	0.00	—	—		
Gastrectomy with any one comorbidity	10812	83	21.9	5.40	(4.17, 7.00)***		
Gastrectomy with any 2 comorbidities	6367	49	25.2	5.54	(4.03, 7.62)***		
Gastrectomy with >2 comorbidities	5416	41	31.0	6.25	(4.42, 8.84)***		

Rate^#^, per 10 000 person-year;

Adjusted HR^†^: multivariate analysis including age and sex.

Adjusted HR^§^: multivariate analysis including age, sex, cholecystitis, diabetes mellitus, and cancer.

*P < 0.05; ***P < 0.001.

^a^CO*P*D = chronic obstructive pulmonary disease; ^b^IBD = Inflammatory Bowel Disease.
